# Biomass Partitioning and Its Relationship with the Environmental Factors at the Alpine Steppe in Northern Tibet

**DOI:** 10.1371/journal.pone.0081986

**Published:** 2013-12-12

**Authors:** Jianbo Wu, Jiangtao Hong, Xiaodan Wang, Jian Sun, Xuyang Lu, Jihui Fan, Yanjiang Cai

**Affiliations:** 1 The Key Laboratory of Mountain Environment Evolution and Its Regulation, Institute of Mountain Hazard and Environment, CAS, Chengdu, China; 2 University of Chinese Academy of Sciences, Beijing, China; Beijing Forestry University, China

## Abstract

Alpine steppe is considered to be the largest grassland type on the Tibetan Plateau. This grassland contributes to the global carbon cycle and is sensitive to climate changes. The allocation of biomass in an ecosystem affects plant growth and the overall functioning of the ecosystem. However, the mechanism by which plant biomass is allocated on the alpine steppe remains unclear. In this study, biomass allocation and its relationship to environmental factors on the alpine grassland were studied by a meta-analysis of 32 field sites across the alpine steppe of the northern Tibetan Plateau. We found that there is less above-ground biomass (M*_A_*) and below-ground biomass (M*_B_*) in the alpine steppe than there is in alpine meadows and temperate grasslands. By contrast, the root-to-shoot ratio (*R:S*) in the alpine steppe is higher than it is in alpine meadows and temperate grasslands. Although temperature maintained the biomass in the alpine steppe, precipitation was found to considerably influence M*_A_*, M*_B_*, and *R:S*, as shown by ordination space partitioning. After standardized major axis (SMA) analysis, we found that allocation of biomass on the alpine steppe is supported by the allometric biomass partitioning hypothesis rather than the isometric allocation hypothesis. Based on these results, we believe that M*_A_* and M*_B_* will decrease as a result of the increased aridity expected to occur in the future, which will reduce the landscape’s capacity for carbon storage.

## Introduction

Biomass allocation was an important character for the process of characterization of plant physiological ecology [1], moreover, it also was the result of the plant long-term adapted to different environmental conditions [2].The Biomass allocation also reflect show photosynthates are allocated between above-ground and below-ground biomass [3]. Biomass allocation above-ground and below-ground affects plant growth as well as the overall function of the ecosystem and biogeochemical cycles [4], [5]. Therefore, the mechanism by which plants respond to variations in the availability of resources in their environment is a major question in plant ecology [6]. Two important hypotheses regarding biomass allocation of plants have been proposed: (i) optimal partitioning and (ii) isometric allocation [2], [7], [8]. The optimal partitioning hypothesis suggests that plants respond to variations in the environment by partitioning biomass among various plant organs to maximize the plants’ growth rate [9], [10]. For example, plants in arid regions are rooted deeper than those in humid environments [11], [12]. On the contrary, the isometric allocation hypothesis predicts the net primary productivity of the roots *vs* the net primary productivity of the shoots (BNPP:ANPP) isometrically without considering the differences in plant species or community types [13]–[15]. Thus far, biomass allocation has been widely examined: investigations have focused on individual organisms as well as whole ecosystems. However, no conclusion about biomass allocation has yet been presented.

Optimal partitioning theory might explain the effect of environmental factors on the allocation of plants’ photosynthetic products, but this theory does not consider the size of the individual plants [8], [16]. The allometric biomass partitioning theory, on the other hand, may resolve biomass allocation patterns in terms of plant size by using standardized major axis (SMA) regression [8], [17]. However, this theory does not provide quantitative descriptions about how environmental factors affect biomass allocation. It also cannot explain the mechanism behind how photosynthates are allocated to different organs [18]. Furthermore, it is still hotly debated whether a uniform biomass allocation pattern is applicable to different ecosystems [19].

The alpine steppe is the largest grassland type in the Tibetan Plateau, which contributes significantly to the global carbon cycle [1]. In the alpine grassland ecosystem, few soil nutrients, aridity, and low temperatures limit plant growth [20], [21]. According to the optimal partitioning hypothesis, environmental factors likely affect how plant biomass is allocated. At the individual plant level, fewer soil nutrients (particularly nitrogen and water) results in an increase in root biomass. On the contrary, root biomass decreases and shoot biomass increases as soil nutrients increase. This partition model is appropriate for different types of vegetation and life forms of plants [22]–[26]. Studies have shown that plants allocate more biomass to their roots when water and nutrients in grassland ecosystems are limited [27], [28]. Moreover, studies have also suggested that plants allocate photosynthates to root in low-temperature environments, which may increase the rate of nutrient absorption and help the plants adapt to environmental conditions [29]–[31]. However, Yang et al. (2009a) reported that on the Tibetan alpine grasslands, the relationship between roots and shoots supports the isometric allocation hypothesis [32]. They also found that this isometric relationship is independent of soil nitrogen and moisture [32]. These results indicate that the mechanism of biomass allocation in the alpine steppe is still misunderstood and unverified in alpine and arid environments. Therefore, this subject requires further investigation. In the present study, we investigated (i) the mechanism behind allocating root and shoot biomass in the Tibetan alpine grassland and (ii) the main factors that affect biomass allocation in the alpine steppe of northern Tibet.

## Materials and Methods

### Collecting Biomass and Soil Samples

**Figure 1:**
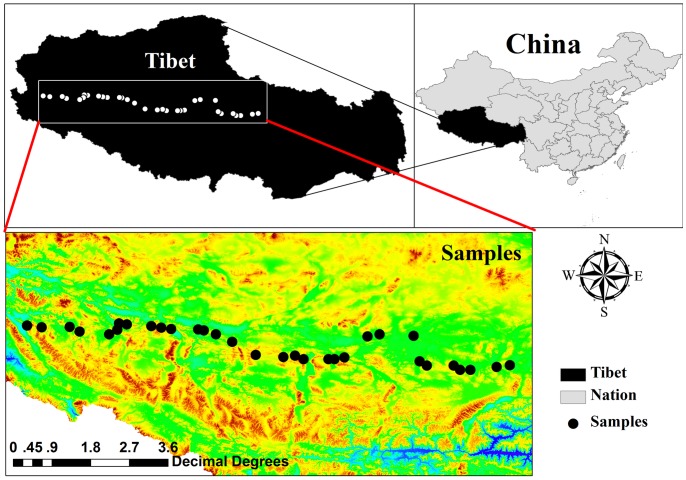
Spatial distribution of the sampling sites in *S. Purpurea* alpine steppe in northern Tibet.

In August 2012, 32 sites were selected on *Stipa purpurea* alpine steppe from Nagqu County to Gar County in northern Tibet. Sampling sites were established at intervals of 30 km (Fig. 1, Table 1). In each site, no specific permits were required for collecting samples and the field studies did not involve endangered or protected species. We selected flat sites with well-protected vegetation. We harvested the aboveground biomass (M*_A_*) and the belowground biomass (M*_B_*) from three blocks of 0.5 m×0.5 m in each site. We collected M*_B_* from soil depths of 0 cm to 15 cm, where most of belowground biomass is located [33], [34].The root samples obtained from the blocks were immediately placed in a cloth bag and then soaked in water to remove the residual soil using a 0.5 mm sieve. Biomass was oven-dried at 65°C until a constant weight was reached, and then it was weighed to the nearest 0.01 g.

**Table 1:** Site description of *S. purpurea* alpine steppe.

Site	County	Dominant species	Mean annualprecipitation (MAP,mm)	Mean annual temperature(MAT,°C)
**S1**	Nakchu	*S. purpurea Kobre siahumilis*	428.1	–1.5
**S2–S7**	Baingoin	*S. purpurea Carex moorcroftii*	321.7	–0.8
**S8**	Xainza	*S. purpurea C.moorcroftii*	304.5	–0.4
**S9–S17**	Nyima	*S. purpurea C.m oorcroftii*	200	–0.4
**S18–S24**	Gêrzê	*S. purpurea*	170.1	0.10
**S25–S30**	Gêgyai	*S. purpurea*	120	0.45
**S31–S32**	Gar	*S. purpurea*	72.1	0.7

Soil samples were collected from two different depths (0–15 cm and 15–30 cm), air-dried, and sieved (2 mm mesh). The fine roots were extracted by hand picking for physical and chemical analyses. The total nitrogen content (TN; TN1∶0–15 cm, TN2∶15–30 cm) of the soil was determined using the micro-Kjeldahl digestion method. The available nitrogen content (AN; AN1∶0–15 cm, AN2∶15–30 cm) of the soil was determined using the alkaline hydrolysis diffusion method. All of the element concentrations were expressed as mg⋅g^−1^ on a dry weight basis.

### Data Analysis

M*_A_* in grasslands can be considered as annual aboveground net primary productivity (ANPP). Blow-ground net primary productivity (BNPP) was calculated using Gill’s method:
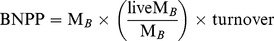
(1)where (live M*_B_*/M*_B_*) = 0.6 and turnover = 0.0009(g⋅m^−2^)×M*_A_* +0.25 [35], [36]. In the present study, the value for (live M*_B_*/M*_B_*) was 0.79, which was measured by Zhou (2001) in the Qinghai region [37]. The relationship between log ANPP and log BNPP was constructed using Model II regression [14], [15]. The slope (α) and y-intercept (log b) of the allocation function were determined by standardized major axis (SMA) tests [38]. The heterogeneity between slopes was determined by performing a permutation test and was rejected if P>0.05 [15]. We analyzed the correlations between environmental factors and the measured M*_A_*, M*_B_*, and root-to-shoot ratios (*R:S*) using the Pearson correlation. We also examined relationships between M*_A_*, M*_B_*, *R:S*, and environmental factors using regression and ordination space partitioning to find the main environmental factors that affected M*_A_*, M*_B_*, and *R:S*. Analyses were performed using SPSS software version 16.0 (IBM; Armonk, NY).

## Results

### Variations in the Chemical Properties of the Soil as well as MA, MB, and R:S

**Figure 2:**
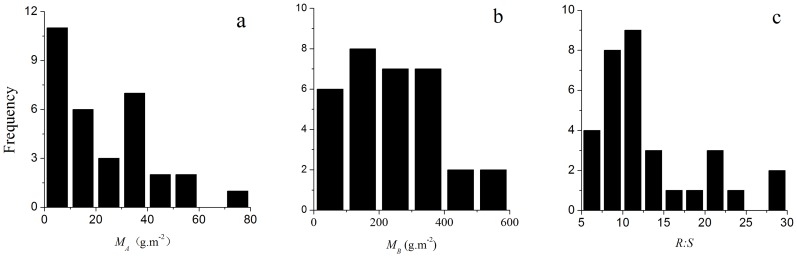
Frequency distributions of (a) above-ground biomass (*M_A_*), (b) below-ground biomass (*M_B_*), and (c) root-to-shoot ratio (*R:S*) in *S. purpurea* alpine steppe.

Small variations in the chemical properties of the soil along the sampled transect were found. There also was not significance in available nitrogen and total nitrogen between the two soil layers (Table 2). We found large variations in M***_A_***, M***_B_***, and *R:S* along the sampled transects (Fig. 2). M*_A_* ranged from 2.32 g⋅m^−2^ to 73.6 g⋅m**^−^** ^2^, while M*_B_* ranged from 22.40 g⋅m^−2^ to 587.32 g⋅m^−2^. *R:S* ranged from 6.19 to 29.15 (Table 3). The median values of M*_A_*, M*_B_*, and *R:S* in *S. purpurea* alpine steppe were 17.16 g⋅m^−2^, 233 g⋅m^−2^, and 11.83, respectively (Table 3).

**Table 2:** Chemical properties of soils in *S. purpurea* alpine steppe.

	Min	Max	Mean	Std. Error	Std. Deviation
**AN1 mg·g** ^−**1**^	0.013	0.110	0.057a	0.004	0.025
**AN2 mg·g** ^−**1**^	0.008	0.095	0.051a	0.004	0.023
**TN1 mg·g** ^−**1**^	0.386	1.630	1.008a	0.061	0.342
**TN2 mg·g** ^−**1**^	0.292	1.921	0.980a	0.064	0.362

**Table 3:** Descriptive statistics of above-ground biomass (*M_A_*), below-ground biomass (*M_B_*), and root-to-shoot (*R*:*S*) ratio in *S. purpurea* alpine steppe.

	*M_A_* (g·m^−2^)			*M_B_* (g·m^−2^)			*R*:*S* ratio		
	Min	Max	Median	Min	Max	Median	Min	Max	Median
Present study	2.32	73.6	17.16	22.4	587.32	233	6.19	29.15	11.83
Yang et al. (2009)	9.8	267.4	42.8	44.6	1934.8	206	0.8	13	5.2

### Biomass Allocation for *S.purpurea* Alpine Steppe

**Figure 3:**
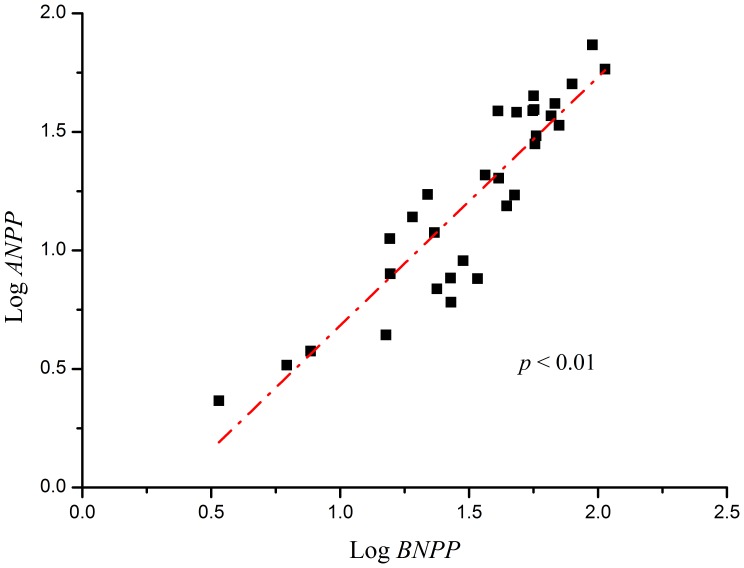
Relationships between above-ground net primary production (ANPP) and below-ground net primary production (BNPP) in alpine steppe by SMA analysis.

The slope (α) of the plotted relationship between log ANPP and log BNPP of *S. purpurea* alpine grasslands was 0.87 with 95% confidence intervals of 0.75 and 1.01 (Fig. 3). The slope (α) was significantly different from the slope obtained from SMA analysis when the isometric hypothesis was used.

### Effects of Soil Nitrogen and Environmental Factors on Biomass and R:S

**Figure 4:**
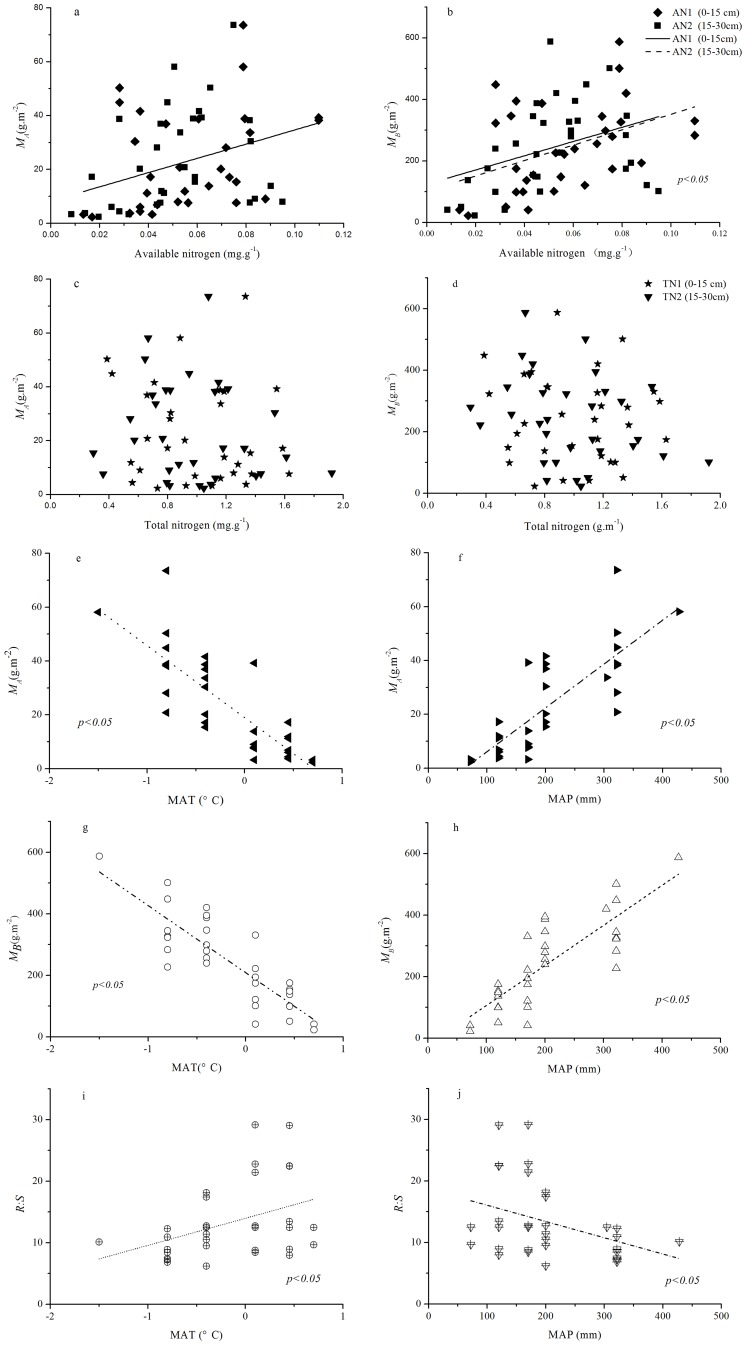
Relationships between biomass allocation (*M_A_*, *M_B_*, and R:S) and environmental factors in alpine steppe. Regressions are shown: (a) *M_A_* versus available nitrogen, (b) *M_B_* versus available nitrogen,(c) *M_A_* versus total nitrogen, (d) *M_B_* versus total nitrogen,(e) *M_A_* versus MAT,(f) *M_A_* versus MAP,(g) *M_B_* versus MAT, (h) *M_B_* versus MAP, (i) *R:S* ratio versus MAT, and (j) *R:S* ratio versus MAP.

**Figure 5:**
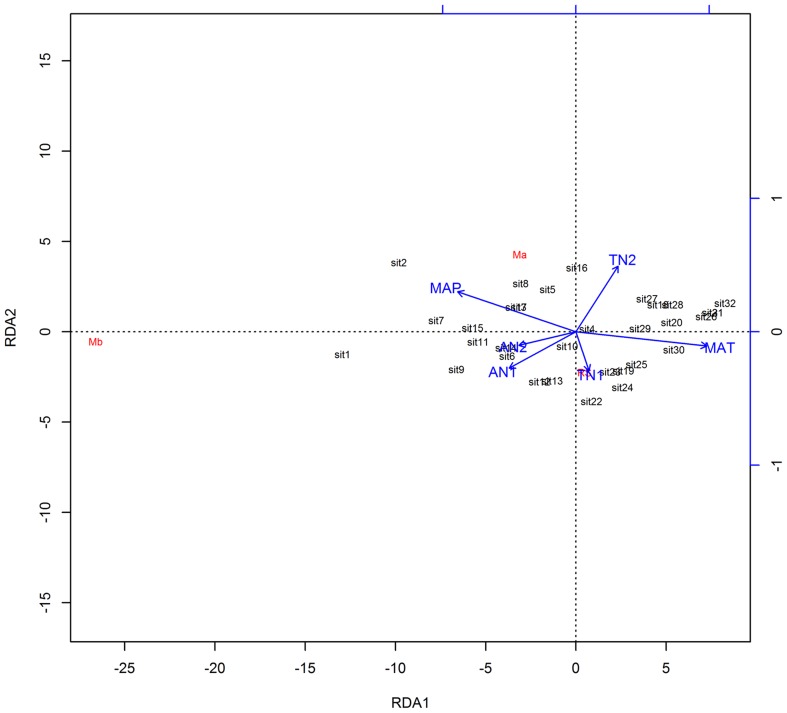
Analysis of the relationship of above-ground biomass (*M_A_*), below-ground biomass (*M_B_*), and root-to-shoot ratio (*R:S*) with the environmental factors by ordination space partitioning method.

Using the Pearson correlation analysis, we found that M***_A_*** and M***_B_*** exhibited a significantly positive correlation with available nitrogen in the soil. However, M***_A_*** and M***_B_*** did not exhibit a significant correlation with total nitrogen (Table 4). The *R:S* ratio also did not exhibit a significant correlation with soil nitrogen (total or available). M***_A_***, M***_B_***, and *R:S* did correlate with the MAP of the sampling sites, while these correlations differed from the ones found with MAT (Table 3). In this study, we found that the regression analysis showed the same results as the Pearson correlation analysis (Fig. 4). Using the ordination space partitioning method, we found that MAP was the main factor that affected M*_A_*, M*_B_*, and *R:S* (Fig. 5).

**Table 4:** Pearson’s correlation between *M_A_*, *M_B_*, and *R*:*S* with the environmental factors.

	AN1(0 to 15 cm)	AN2(15 to 30 cm)	TN1(0 to 15 cm)	TN2(15 to 30 cm)	MAT(°C)	MAP(mm)	
*M_A_*	0.351*	0.317	–0.126	–0.165	–0.809**		0.791**
*M_B_*	0.429*	0.372*	–0.088	–0.285	–0.853**		0.817**
*R*:*S*	–0.047	–0.083	0.207	–0.082	0.392*		–0.378*

Correlation is significant at the 0.01 level (two-tailed).

Correlation is significant at the 0.05 level.

## Discussion

### M_A_, M_B_, and R:S in the Alpine Steppe

In the present study, amounts of M***_A_*** and M***_B_*** in the alpine steppe (mean = 23.20 g⋅m^–2^) were found to be lower than those in the alpine meadows [32] and in the temperate grasslands of China [39]. By contrast, *R:S* in the alpine steppe was found to be higher than it is in China’s alpine meadows [32] and temperate grasslands [39] as well as in temperate grasslands of other regions [1]. These results show that precipitation and temperature affect plant growth and biomass allocation [1], [40]. Slower root turnover in colder environments might also results in higher R:S ratios [41]–[43]. M*_A_*, M*_B_*, and *R:S* values found in the present study are not consistent with results reported by Yang et al. (2009a), who performed a field investigation from 2001 to 2004 [32]. *R:S* values have the potential to vary greatly as a result of climate change and anthropogenic activities [44]–[48].

### Mechanism of Biomass Allocation in the Alpine Steppe

Based on the results of our SMA analysis, we found that biomass allocation on the alpine steppe does not fit the isometric hypothesis. By contrast, Yang et al. (2009a) previously reported that biomass allocation on the alpine steppe is supported by the isometric allocation hypothesis [32]. In the harsh alpine ecosystem, scarce precipitation and low temperatures allow plants to allocate more biomass to the roots, which helps plants survive [29]–[31]. Moreover, roots have also been found to store carbohydrates in alpine grasslands [49], [50]. Therefore, biomass allocation in the alpine steppe may reflect the allometric biomass partitioning hypothesis rather than the isometric allocation hypothesis.

### Relationships between Environmental Factors and MA, MB, and R:S

Precipitation and temperature are considered to be the limiting factors for the growth and distribution of vegetation over the long term [51], [52]. In the present study, M*_A_*, M*_B_*, and *R:S* were mainly affected by the environmental factor of precipitation (MAP), as revealed by ordination space partitioning analysis. These results are consistent with those of other reports about the alpine steppe [46], [53]–[56]. The low temperature in the growing season did not limit the growth of alpine plants because these plants shave evolved to survive in the cold alpine climate [57]. The amounts of aboveground and belowground biomass are higher in sites with higher humidity, but the MAT is also relatively low on the alpine steppe. Precipitation is an essential factor that controls the functions of ecosystems in terrestrial biomes, particularly in arid and semiarid ecosystems [58]. Therefore, precipitation is the main factor that influences amounts of biomass in the alpine steppe.

Moreover, in the present study, we found that amounts of M*_A_* and M*_B_* on the alpine steppe were affected by the available nitrogen content in the soil but not by the total nitrogen content of the soil. These results are inconsistent with those from previous studies, which have showed that M*_A_* and M*_B_* are positively related to total nitrogen content [32], [59]. Because available nitrogen can be used to approximate the relative supply of nutrients, nitrogen may be another factor that controls ecosystem processes in regions with abundant water resources [60].

## Conclusion

As the climate changes, the degree of aridity has been consistently increasing in northern Tibet [61]. Changes in biomass allocation on the alpine steppe are likely to affect the carbon cycle and the general functioning of the alpine ecosystem. In the present study, we found that the *R:S* ratio in the alpine steppe was higher than that of other grassland systems. The amounts of aboveground and belowground biomass as well as the *R:S* ratio were primarily affected by precipitation. The observed biomass allocation was found to follow the allometric biomass partitioning theory rather than the isometric allocation hypothesis. These results suggest that the landscape’s capacity to store carbon will potentially decrease as the degree of aridity in northern Tibet increases.
